# South Side Views

**Published:** 1884-08

**Authors:** 


SOUTHSIDE VIEWS.—Dr. Whedon and the Fathers, also Dr. Haygood's "Our Brother in Black." By Rev. W. J. Scott, North Georgia Conference. Price, 25 cents. JAS. P. HARRISON & Co., Publishers, Atlanta, Ga. This is a handsomely printed pamphlet of 80 pages, in which the author reviews at some length the book of Dr. Haygood, "Our Brother in Black," and gives his own views on the negro problem. It is well worth reading.

